# Membrane Phenotypic, Metabolic and Genotypic Adaptations of *Streptococcus oralis* Strains Destined to Rapidly Develop Stable, High-Level Daptomycin Resistance during Daptomycin Exposures

**DOI:** 10.3390/antibiotics12071083

**Published:** 2023-06-21

**Authors:** Nagendra N. Mishra, Rodrigo de Paula Baptista, Truc T. Tran, Christian K. Lapitan, Cristina Garcia-de-la-Maria, Jose M. Miró, Richard A. Proctor, Arnold S. Bayer

**Affiliations:** 1 Division of Infectious Diseases, The Lundquist Institute at Harbor-UCLA Medical Center, 1124 West Carson St. MRL Bldg. Room 224, Torrance, CA 90502, USA; 2 The David Geffen School of Medicine, University of California, Los Angeles (UCLA), Los Angeles, CA 90095, USA; 3 Center for Infectious Diseases, Houston Methodist Research Institute, Houston, TX 77030, USA; 4 Division of Infectious Diseases, Department of Medicine, Houston Methodist Hospital, Houston, TX 77030, USA; 5 Department of Medicine, Weill-Cornell Medical College, New York, NY 10065, USA; 6 Infectious Diseases Service, Hospital Clinic—IDIBAPS, University of Barcelona, 08036 Barcelona, Spain; 7 CIBERINFEC, Instituto de Salud Carlos III, 28220 Madrid, Spain; 8 The Department of Medicine, University of Wisconsin School of Medicine, Madison, WI 53705, USA

**Keywords:** high-level daptomycin resistance, *S. oralis*, lipids, glycolysis

## Abstract

The *Streptococcus mitis-oralis* subgroup of viridans group streptococci are important human pathogens. We previously showed that a substantial portion of *S. mitis-oralis* strains (>25%) are ‘destined’ to develop rapid, high-level, and stable daptomycin (DAP) resistance (DAP-R) during DAP exposures in vitro. Such DAP-R is often accompanied by perturbations in distinct membrane phenotypes and metabolic pathways. The current study evaluated two *S. oralis* bloodstream isolates, 73 and 205. Strain 73 developed stable, high-level DAP-R (minimum inhibitory concentration [MIC] > 256 µg/mL) within 2 days of in vitro DAP passage (“high level” DAP-R [HLDR]). In contrast, strain 205 evolved low-level and unstable DAP-R (MIC = 8 µg/mL) under the same exposure conditions in vitro (“non-HLDR”). Comparing the parental 73 vs. 73-D2 (HLDR) strain-pair, we observed the 73-D2 had the following major differences: (i) altered cell membrane (CM) phospholipid profiles, featuring the disappearance of phosphatidylglycerol (PG) and cardiolipin (CL), with accumulation of the PG-CL pathway precursor, phosphatidic acid (PA); (ii) enhanced CM fluidity; (iii) increased DAP surface binding; (iv) reduced growth rates; (v) decreased glucose utilization and lactate accumulation; and (vi) increased enzymatic activity within the glycolytic (i.e., lactate dehydrogenase [LDH]) and lipid biosynthetic (glycerol-3-phosphate dehydrogenase [GPDH]) pathways. In contrast, the 205 (non-HLDR) strain-pair did not show these same phenotypic or metabolic changes over the 2-day DAP exposure. WGS analyses confirmed the presence of mutations in genes involved in the above glycolytic and phospholipid biosynthetic pathways in the 73-D2 passage variant. These data suggest that *S. oralis* strains which are ‘destined’ to rapidly develop HLDR do so via a conserved cadre of genotypic, membrane phenotypic, and metabolic adaptations.

## 1. Introduction

The viridans group streptococci (VGS), particularly the *S. mitis/oralis* subgroup, are important human pathogens in a variety of invasive endovascular infections, including “the toxic Strep shock syndrome” in neutropenic cancer patients and infective endocarditis (IE) [1,2,3,4,5]. The treatment of *S. mitis/oralis* infections has become difficult due to the relatively high prevalence of penicillin (~25%) and cephalosporin resistance, including to third generation agents [1]. In addition, *S. mitis*/*oralis* strains can exhibit vancomycin tolerance, further limiting therapeutic choices [6]. DAP has been proposed as a plausible alternative for severe infections caused by β-lactam-resistant *S. mitis/oralis* strains. However, Garcia-de-la-Maria et al. reported that >25% of ~100 DAP-susceptible (DAP-S) clinical bloodstream *S. mitis/oralis* strains were ‘destined’ to rapidly develop stable, high-level DAP-R (DAP MIC > 256 µg/mL) within 2 days of DAP exposure; this phenomenon has been confirmed by others in vitro and in simulated ex vivo IE models, as well as in vivo in experimental IE models [1]. Such rapid development of stable, rapid, and high-level DAP-R (HLDR) evolving upon DAP exposures has been rarely observed in other clinically important gram-positive pathogens (e.g., *S. aureus* and enterococci) [1,7].

We have previously reported on a plethora of potential mechanisms underlying HLDR in *S. mitis/oralis* [8,9,10], especially linked to key cell membrane (CM) events, including: selective and potentially ‘altruistic’ DAP hyper-accumulation among DAP-R cells; altered fluidity; enhanced surface charge; perturbations of key phospholipids (PLs) involved in DAP docking and its mechanism of action (i.e., PG and CL, correlated with mutations in *cdsA* or *pgsA*); and metabolic perturbations (e.g., in the glycolytic pathway) [11].

The current study was designed to build upon these latter studies, and specifically catalogue the above phenotypic perturbations, using two recent clinical isolates which appear destined to evolve either the HLDR or non-HLDR phenotype early (<2 d) following DAP exposures. In addition to the membrane and metabolic phenotypes noted above, we also assessed potential accumulations of relevant mutations during such short-term DAP passage in HLDR vs non-HLDR strains by whole genome sequencing (WGS).

Note: The term “DAP-resistance” (DAP-R) was used throughout the manuscript instead of “DAP-nonsusceptibility” for a more facile presentation.

## 2. Materials and Methods

### 2.1. Bacterial Strains

Two recent DAP-S clinical *S. oralis* bloodstream isolates (73 and 205) from patients with IE who were unexposed to DAP therapy were studied [1] (Table 1). These two strains were identified as *S. mitis/oralis* by MALDI-TOF VITEK^®^ MS Biomerieux- Marcy-l’Étoile, France. MALDI-TOF cannot distinguish these two subspecies [12]; their subspeciation as *S. oralis* were defined by whole genome sequencing using metaphlan v3.1.0 [13].

### 2.2. In Vitro DAP Passage

Both parental study isolates were subjected to short-term (2 d) in vitro DAP passage to select for DAP-R variants. Each DAP-S parental strain was cultured overnight in BHI broth (BHIB). An initial inoculum of OD_600_ = 1.00 [~10^8^ CFU/mL] was then exposed to 20 µg/mL of DAP in BHIB + 50 µg/mL CaCl_2_. Surviving colonies were serially passaged for a 2-day period under the same conditions as above. Surviving colonies after each day’s passage were collected and stored at −80 °C for subsequent MIC testing. The 2 d post-passage isolates were then serially re-passaged for 5 days in antibiotic-free BHI media to determine the stability of DAP-R [8,9,10]. The DAP MICs of these DAP-passage and antibiotic-free post-passage strains were determined by broth microdilution assay (see below). A minimum of three independent experiments were performed on separate days to analyze the MIC data.

### 2.3. Minimum Inhibitory Concentrations (MICs)

DAP was obtained from Merck & Co., Inc. (Whitehouse Station, NJ, USA). DAP MIC testing was carried out by the CLSI-recommended broth microdilution techniques, with 50 µg/mL of CaCl_2_ added to BHIB (Difco, Franklin Lakes, NJ, USA). In addition, BHI agar supplemented with 5% lysed horse blood (Difco, Franklin Lakes, NJ, USA) was used to quantify agar plate colony counts. There are no formal CLSI-recommended DAP breakpoints for VGS strains; however, streptococcal strains with DAP MICs ≥ 2 µg/mL are considered as DAP-R [1,8,9,10]. A minimum of three independent runs were performed on different days, with the average MIC reported herein.

### 2.4. Phenotypic Assays

#### 2.4.1. Surface Charge

The relative positive cell surface charge of the study strains was determined using the standard cytochrome C (Cyt C) binding assay as described previously [8,9]. Briefly, stationary phase cells were pelleted by centrifugation, washed with MOPS (3-morpholinopropane1-sulfonic acid) buffer (pH 7.0), resuspended in the same buffer at OD_578_ ≈ 1.0, and incubated with 0.5 mg/mL of the highly cationic Cyt C for 10 min. Then, the residual quantity of Cyt C remaining in the bacterial supernatant was determined spectrophotometrically at OD_530_ nm [8,9]. A decrease in the quantity of Cyt C binding (i.e., more cation in the supernate) equates to a greater positively charged bacterial surface [8,9]. The quantified data are presented as an average (±SD) of unbound Cyt C. A minimum of three independent experimental runs were performed on different days. Further, as a confirmatory surface charge quantification, a fluorescein isothiocyanate (FITC)-labeled poly-l-lysine (PLL) binding assay was performed using flow cytometry (FACS Calibur^®^; Beckman Instruments, Alameda, CA, USA), as reported before [8,9]. Data were represented as mean fluorescent units ± SDs. Decreased PLL binding was correlated with the more positively charged *S. oralis* surface [8,9]. At least three independent runs were performed on separate days.

#### 2.4.2. CM Fluidity

Bacterial strains were grown in BHIB overnight at 37 °C, washed with PBS and adjusted to an OD_600_ = 1.0 (≈10^8^ CFU/ mL) as before. Fluidity measurements were carried out by using the fluorescent probe, 1,6-diphenyl-1,3,5-hexatriene (DPH; excitation and emission wavelengths = 360 nm and 426 nm, respectively). Polarization spectrofluorimetry was used to quantify fluorescence polarization (horizontal vs vertical) and to define the polarization index (PI) value as before (LS50 Perkin-Elmer, Valencia, CA, USA) [8,9]. An inverse correlation exists between PI value and fluidity (i.e., a lower PI value equates to a higher extent of CM fluidity) [8,9]. Three independent experiments were conducted on separate days.

#### 2.4.3. CM Phospholipid (PL) Composition

The protocol for PL extractions has been described elsewhere [8,9]. *S. mitis/oralis* CMs consist of three major PLs, i.e., phosphatidic acid (PA; the major precursor molecule within the cardiolipin [CL] biosynthetic pathway [8,9]) phosphatidylglycerol (PG); and cardiolipin (CL). These three major PLs were separated, and their relative proportionality determined by two-dimensional thin-layer chromatography (2D-TLC), using a unique solvent system as detailed before [8,9]. All PL spots on TLC plates were identified (as compared to known PL standards) using iodine vapor exposure and by spraying with CuSO_4_ (100 mg/mL) containing 8% phosphoric acid (*v*/*v*) and heated at 180 °C [8,9]. Each identified PL spot from TLC plates was scraped and digested at 180 °C for 3 h with 0.3 mL 70% perchloric acid into the inorganic form of phosphate. The relative biochemical quantification of each PL was determined spectrophotometrically at OD_660_ [8,9]. Data represented the mean (±SD) percentages of the three major PLs (PA + PG + CL = 100%). All *S. mitis oralis* strains contain a major glycolipid species visible on TLC plates, the analysis of which is not included in our quantifications [8,9]. A minimum of three independent experiments were performed on separate days.

#### 2.4.4. Lipidomics Analyses by Mass Spectrometry

To confirm the above 2D-TLC assays, as well as to identify additional PLs-of-interest, formal lipodomic assays were carried out. Pelleted and washed cells from overnight growth of the study strains (parental and 2-day DAP passage) were transferred to extraction tubes with PBS. A modified Bligh and Dyer extraction method was used to extract lipid samples [14]. Prior to extraction, an internal standard mixture consisting of 70 lipid standards across 17 subclasses was added to each sample (AB Sciex 5,040,156, Avanti 330,827, Avanti 330,830, Avanti 330,828, Avanti 791,642). Following two consecutive extractions, pooled organic layers were dried down in a Thermo Speed Vac SPD300DDA using ramp setting 4 at 35 °C for 45 min with a total run time of 90 min. Lipid samples were then resuspended in 1:1 methanol/dichloromethane with 10 mM ammonium acetate and transferred to robovials (Thermo 10,800,107) for analysis. Samples were analyzed by direct infusion on a Sciex 5500 with a Differential Mobility Device (DMS) (comparable to a Sciex Lipidyzer platform) with a targeted acquisition list consisting of 1450 lipid species across 17 subclasses. The DMS was tuned with EquiSPLASH LIPIDOMIX (Avanti 330,731). Data analysis was performed with in-house data analysis workflow. Instrument settings, MRM lists, and analysis methods are available [15]. Quantitative values were normalized to cell counts. Pilot lipidomic analyses have identified several key lipid species in *S. mitis oralis* strains in addition to PG and PA, including diacylglycerol (DAG), triacylglycerol (TAG), phosphatidylcholine (PC), phosphatidylethanolamine (PE), phosphatidylinositol (PI), phosphatidylserine (PS), free fatty acids (FFAs), ceramides (Cer d18:1), dihydroceramides (Cer d18:0), hexosylceramides (HexCER), lactosylceramides (LacCER), cholesterol esters (CE), and sphingomyelin (SM). The Sciex 5500 lipidomics platform was not able to detect the CL metabolite because of its higher mass, as well as the detection limit of the instrument.

#### 2.4.5. Quantification of DAP Binding

Binding of DAP to whole cells was assessed spectrofluorometrically, using a fluorescent BODIPY-DAP binding assay [16]. A bacterial inoculum of 1.0 × 10^6^ CFU/mL was exposed to 5 μM of BODIPY-DAP, and then incubated in the dark at room temperature for 20 min. BODIPY-DAP fluorescence intensity was measured employing spectrofluorometry (excitation wavelength = 488 nm; emission wavelength = 530 nm). Because the antimicrobial potency of BODIPY-tagged DAP is ~4-fold lower than that of standard, unbound DAP, the DAP exposure concentrations used for these binding assays were calculated to be ~4-fold higher than the average free human serum therapeutic DAP level of 3.6 ug/mL [16].

### 2.5. Metabolic Assays

We have previously reported on the relationship of perturbations within the glycolytic pathway and DAP-R in *S. mitis/oralis* strains [11]. We, thus, carried out a series of assays relative to this pathway to further define these metrics in our parental vs. 2-day DAP-passage variants. These assays involved both measurements of key biometabolites and quantitative enzymatic activity relative to the glycolytic pathway.

#### 2.5.1. Cultivation Conditions

For selective metabolic and biochemical assays below, study isolates were grown in BHIB supplemented with 2 µg/mL D-glucose (BHI + glucose) or on blood agar plates (VWR Scientific, Radnor, PA, USA). For all studies, *S. oralis* pre-cultures were inoculated (1:10) from overnight cultures into 50 mL of BHI in 50 mL conical tubes and incubated at 37 °C without shaking for 4 h.

#### 2.5.2. Measurement of Growth Rates

The exponential growth phase pre-cultures of *S. oralis* strains were collected by centrifugation at 4000 rpm at 22 °C and suspended in 1 mL of culture medium. Primary cultures were inoculated into 50 mL (BHI +/− glucose) within 50 mL conical tubes to an absorbance at 600 nm (A_600_) of 0.16 and incubated at 37 °C without shaking. *S. oralis* strain cultures were mixed by inversion every 30 min prior to sampling. The growth rate (A_600_) of the *S. oralis* strains were recorded every 60 min for 5 h. Based on our prior published time-dependent metabolic outcome metrics in other *S. mitis/oralis* strains [11,17], we selected these early growth time-points for evaluating growth rates, as well as for the metabolic and biochemical assays outlined below [11,17].

#### 2.5.3. Determination of Glucose, Lactate, Ammonium, and Acetate Concentrations in Cultivation Media

Cell-free media were harvested hourly during the growth rate studies above by centrifugation, transferred to 1.5 mL microcentrifuge tubes, and stored at −20 °C until further use. The above four glycolytic metabolite concentrations in the hourly culture media were measured from three biological replicates each, using their respective quantification kits purchased from R-Biopharm (Washington, MO, USA). Three independent assays were carried out to measure these biometabolite concentrations.

#### 2.5.4. Lactate Dehydrogenase (LDH) and Glycerol-3-Phosphate Dehydrogenase (GPDH) Enzymatic Assays

Strains were cultivated as detailed above, and cells were then collected hourly by centrifugation and disrupted using Fast Prep (FP120 Thermo Savant). Cell debris in the supernates was discarded after centrifugation. The cell-free lysates were then utilized to measure the enzymatic activity of LDH and GPDH after suspension in 100 µL LDH assay buffer (Sigma-Aldrich MAK066 Kit, Burlington, VT, USA). Activities of LDH and GPDH were determined from three independent biological replicates. Protein concentrations were measured using the Bradford protein assay (Fisher Scientific, Waltham, MA, USA).

### 2.6. Genotypic Assay

#### 2.6.1. Whole Genome Sequencing

Prior to DNA extraction for sequencing, bacterial inocula were prepared by growing the bacterial cultures overnight in 10 mL of Brain Heart Infusion broth at 37 °C with agitation. A total of 2 mL of the overnight bacterial cultures were then harvested by centrifugation at 10,000× *g* for 10 min, and the resulting bacterial pellets were washed twice with sterile phosphate-buffered saline (PBS) to remove any residual medium. The bacterial pellets were then resuspended in lysis buffer provided in the kit and incubated at 56 °C for 30 min with gentle shaking to facilitate cell lysis and release of DNA. Subsequently, DNA was purified and eluted using the QIAGEN DNeasy Blood and Tissue Kit (QIAGEN Inc., Valencia, CA, USA) according to the manufacturer’s instructions. The extracted DNA was quantified using a spectrophotometer and stored at −20 °C until further use. Mutations are the eventual source of heritable variation for evolution. Emergence of DAP-R in these strains upon DAP exposures might be related to selecting out pre-existing DAP-R sub-populations, rather than the acquisition of DAP-R genes. In this study, the identification of mutations was carried out using Snippy v.4.6.0, a rapid haploid variant calling pipeline. To minimize the inclusion of false variant calls, several filtering parameters were applied to the samples. These parameters included: (i) minimum coverage of 10 reads; (ii) minimum variant call quality of 100; (iii) minimum map quality of 60 to be sure that the read was uniquely mapped, avoiding potential duplicates; and (iv) minimum quality for the nucleotide was 20, representing an error probability of ~1%.

#### 2.6.2. Genome Assembly

ONT libraries were prepared using the rapid barcode sequencing kit (RBK110.96), and Illumina libraries were prepared using the Illumina Nextera Flex DNA library kit (Illumina, San Diego, CA, USA). Samples were sequenced in both Oxford Nanopore MinION mk1c long-read (ONT) and Illumina NextSeq 2000 150PE short-read sequencing platforms. After sequencing, all reads were trimmed for quality: (i) Q > 8 for ONT reads; and (ii) Q > 25 for Illumina short reads using Guppy basecaller v6.4.8 and Trimmomatic v0.39 [18]. Genome assembly was first performed using ONT-only reads using Flye v2.9 [19] with default parameters. Resulting draft genomes were then submitted to a base-call error correction polishing step using NextPolish v1.4.1 [20]. Genome statistics were calculated by QUAST v5 [21].

#### 2.6.3. Taxon Identification

All trimmed reads were submitted to Metaphlan v3.1.0 [13] for taxon identification using the rel_ab_w_read_stats flag to profile relative abundances and estimate the number of reads coming from each clade if a mixed sample was found. The results were then visualized using Krona v.2.8.1 [22].

#### 2.6.4. Resistome

Assembled genomes were submitted to Abricate v1.0.1 (https://github.com/tseemann/ABRicate, accessed on 5 April 2023) to check for the potential presence of resistance genes in each sample. Variant analysis. Raw short-reads from each sample generated in the Illumina machine were first trimmed for quality > Q25. They were then submitted to snippy v4.4.5 (https://github.com/tseemann/snippy, accessed on 5 April 2023) using as reference the *S. oralis* ATCC 35037 genome (GCF_900637025.1). The resulting vcf file was then annotated using a custom database from the same used reference in SnpEff v4.3 [23]. Tables were generated using Snpsift and compared using custom bash scripts and visualized in Venny v2.1 (https://bioinfogp.cnb.csic.es/tools/venny/, 5 April 2023).

### 2.7. Statistical Analysis

Means and standard deviations (SDs) were determined for all variables. Differences between CM phenotypic and metabolic biochemical assay metrics were analyzed using the two-tailed Student *t* test. *p* values of ≤0.05 were considered as ‘significant’.

## 3. Results

### 3.1. CM Phenotypic Characteristics of HLDR vs. Non-HLDR S. oralis Strains

#### 3.1.1. Emergence of HLDR and Non-HLDR Variants by In Vitro DAP Passage

DAP-S parental strains 73 and 205 were passaged in vitro in DAP for 2 d to derive DAP-R variants (DAP MIC ≥ 2 µg/mL). Strain 73 post-passage (73-D2) exhibited a DAP MIC > 256 µg/mL; in contrast, strain 205 post-passage (205-D2) had a DAP MIC = 8 µg/mL (Table 1). We termed these two strains, HLDR and non-HLDR, respectively. Further, both strains 73-D2 and 205-D2 were evaluated for the stability of DAP-R, following serial passage in DAP-free media for 5 days. Strain 73-D2 showed a stably high DAP MIC (i.e., MIC > 256 µg/mL), while strain 205-D2 exhibited an unstable DAP MIC (i.e., MIC was reduced to 2 µg/mL following passage in antibiotic-free media). Of interest, when each parental strain was passaged in vitro in DAP for a total of 10 d, both strains 73 and 205 evolved high-level (MIC > 256 ug/mL) and stable DAP-R (data not shown).

#### 3.1.2. Cell Surface Charge

DAP-R *S*. *aureus*, enterococci, and some *S. mitis/oralis* strains often exhibit a relatively more positive surface charge than their respective DAP-S parental strains [8,9,16,24,25]. Surprisingly, moderately decreased amounts of cytochrome C were observed in the 73-D2 vs. 73 parental strain supernates, indicating a potentially decreased surface positive charge; in contrast, this phenotype was unaltered in comparing the 205-D2 vs its parental strain (Table 2). The PLL binding assay was performed to confirm the above surface charge data of these strain-sets; the net surface charge by this assay did not significantly differ among passage variants vs. their respective parental strains (data not shown). Thus, taken together, passage in DAP did not appear to substantially impact surface charge in either strain-pair.

#### 3.1.3. CM Fluidity

As shown in Table 2**,** the DAP-R 73-D2 strain displayed significantly more fluid CMs vs. its DAP-S 73 parental strain. In contrast, the 205 strain-pair did not differ in CM fluidity metrics.

#### 3.1.4. DAP Binding

Whole cell DAP binding assays showed modestly higher binding in both DAP-R strains, although only reaching significance for the 73 strain-pair (Table 2).

#### 3.1.5. Quantitative Measurements of PL Contents by 2D-TLC and Lipidomics Analyses

As reported before for other *S. mitis/oralis* strains [8,9,10,25], three major PL species (PG, CL, and PA) were identified by 2D-TLC in both DAP-S 73 and 205 parental strains; CL was the dominant species in both DAP-S parental strains. In the DAP-R 73-D2 strain, PG and CL were undetectable, with only the PG-CL pathway precursor, PA, detected (Table 3; Supplementary Figure S1). In contrast, the 205 strain-pair did not show major alterations in PG and CL content, with only a modest enhancement of PA content (Table 3; Supplementary Figure S1).

**Figure 1:**
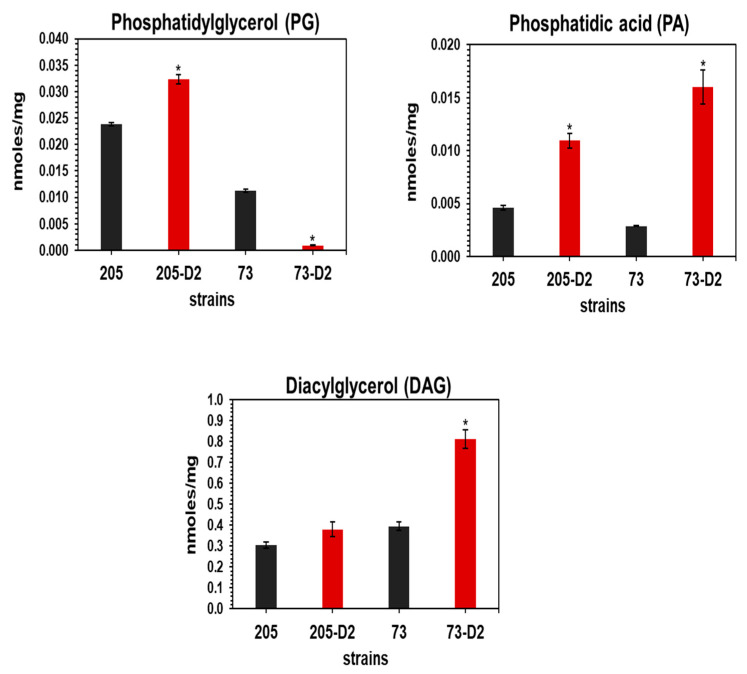
Mass spectrometric-based lipidomic analysis of study strains. These data represent the mean (±SD) of three independent experiments from different lipid extracts. Statistical differences for D2 strains relative to their HLDR and non-HLDR parental strains were carried out by Student’s *t*-test; * *p* < 0.05 Parental strains vs. D2 strains.

Further, mass spectrometry-based lipidomic analyses confirmed these alterations of PL patterns appearing on 2D-TLC and identified additional lipid species-of-interest differentiating these two strain-sets. Thus, similar to the 2D-TLC assays, lipidomic analysis confirmed a major alteration of PL profiles (particularly, in terms of very reduced PG levels, with a compensatory increase in levels of the precursor molecule, PA) in the 73-D2 vs. its 73 parental strain. Moreover, paralleling the 2D-TLC assays in comparing the 205-parental and 205-D2 strain-pair, both the PG and PA species contents were present and readily quantifiable (Figure 1). It should be noted that our lipidomic analysis was not able to identify and quantity CL, due to the limit of detection of our instrument related to the higher degree of mass value of this PL species. However, since CL is a dimer of the PG molecule, it is reasonable to assume that the near-disappearance of PG on lipidomics would translate closely into a very reduced CL content in the 73-D2 strain (as seen in the 2D-TLC assays).

Diacylglycerol (DAG) is the principal intermediate of the CL biosynthetic pathway (Supplementary Figure S3). Increased DAG content was observed in 73-D2 vs. its 73 parental strain, but it was not significantly altered in the 205 strain-pair. Lipidomics also revealed significantly reduced content of several other lipid species (i.e., phosphatidyl ethanolamine [PE], ceramides, sphingomyelin [SM], and free fatty acids [FFA]) in 73-D2 vs. the parental 73 strain; the patterns of these lipid species were not significantly altered in the 205 strain-set. In addition, increased content of phosphatidyl serine (PS) was observed in the 73-D2 vs. the 73 parental strain, but not in the 205 strain-pair. Of interest, phosphatidylcholine (PC) content was almost negligible in the 73 strain-pair, but it was increased in the 205-D2 vs. 205 parental strain (Supplementary Figures S2A–C).

## 4. Metabolic-Biochemical Profiles of HLDR Vs. Non-HLDR *S. oralis* Strains

### 4.1. Growth Profiles

**Figure 2:**
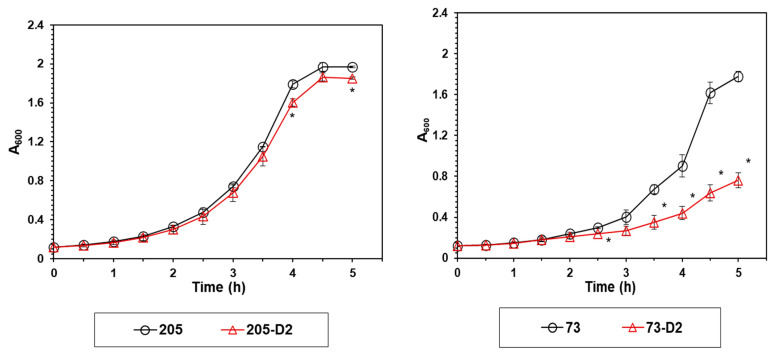
Growth profile of the 73-D2 and 205-D2 strains vs. their respective parental *S. oralis* strains. Growth (A_600_) patterns of D2 strains relative to their HLDR and non-HLDR parental strains were assessed hourly. Data represent the mean (±SD) of three independent cultures grown on different days. Statistical differences for D2 strains vs. their respective HLDR and non-HLDR parental strains were determined by Student’s *t*-test; * *p* < 0.05 Parental strains control vs. D2 strains.

The transition from DAP-S to DAP-R on DAP exposures has been previously correlated with alterations in growth rates and growth yields in both *S. aureus* and selected *S. mitis-oralis* strains [11,26]. We, thus, measured the early growth profiles of our strain-pairs (Figure 2). As expected, the 73-D2 strain exhibited a significantly decreased early growth rate vs. its 73 parental strain (between 3 and 5 h) (Figure 2); such differences were not observed in comparing early growth curves of the 205 strain-pair (Figure 2).

#### 4.1.1. Glucose/Pyruvate Catabolism

**Figure 3:**
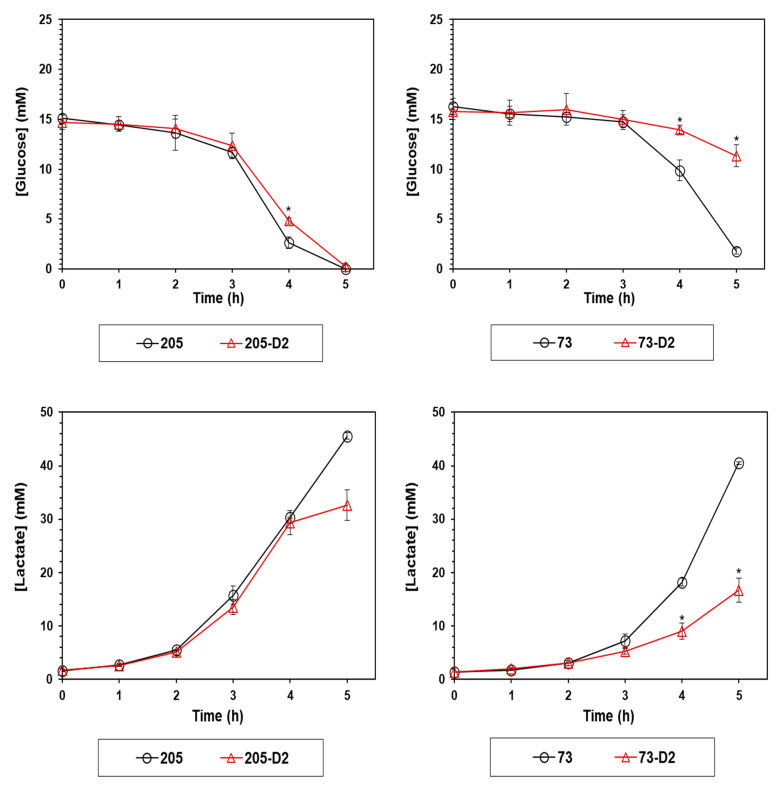
Glucose depletion and lactate accumulation in 73-D2 and 205-D2 vs. their respective parental *S*. *oralis* strains. Glucose depletion and lactate accumulation in D2 strains vs. their respective HLDR and non-HLDR parental strains were analyzed hourly. Data represent the mean (±SD) of three independent biological replicates. Statistical differences for D2 strains vs. their respective HLDR and non-HLDR parental strains were determined by Student’s *t*-test; * *p* < 0.05 Parental strains vs. D2 strains.

Prior studies have linked the emergence of DAP-R in *S. mitis/oralis* strains with perturbations in the glycolytic pathway [11]. To investigate if the glycolytic pathway signatures of the DAP-R 73-D2 vs. 205-D2 strains differed (vs. their respective parental strains), both glucose consumption and lactate accumulation were quantified over the same early growth period as noted above (0–5 h; Figure 3). As expected, the DAP-R 73-D2 strain utilized less glucose than its parental 73 strain, coincident with its relatively slower early growth rates (Figure 3); this was accompanied by decreased lactate accumulation in the culture medium over this same time period (Figure 3). In contrast, neither glucose utilization nor lactate accumulation were altered significantly in comparing the DAP-R 205-D2 vs. its parental 205 strain over this same time period.

#### 4.1.2. Lactate Dehydrogenase (LDH) Activity

**Figure 4:**
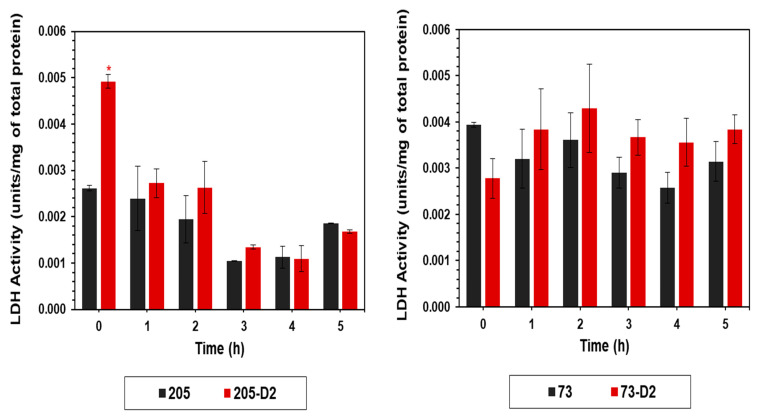
Temporal quantitation of LDH activity of *S. oralis* strains 73-D2 and 205-D2 vs. their respective parental *S*. *oralis* strains. LDH activity was assayed from cell-free lysates from cultures cultivated in BHI and harvested at multiple time points (0–5 h). The data represent the mean (±SD) of three independent experiments. Statistical significance (*) was assessed by using Student’s *t*-test (*p* ≤ 0.05); * *p* < 0.05, D2 strains vs. their respective HLDR and non-HLDR parental strains.

In the glycolytic pathway, the end-product is pyruvate, which in *S. mitis/oralis* strains is then further catabolized by membrane-associated LDH into lactate [27]. Although not reaching statistical significance, there was an obvious trend towards increasing LDH activity during the early growth period (2–5 h) in the DAP-R 73-D2 strain vs. its parental DAP-S 73 strain, which was not observed in the 205 strain-pair (Figure 4).

#### 4.1.3. Glycerol-3-Phosphate Dehydrogenase (GPDH) Activity

**Figure 5:**
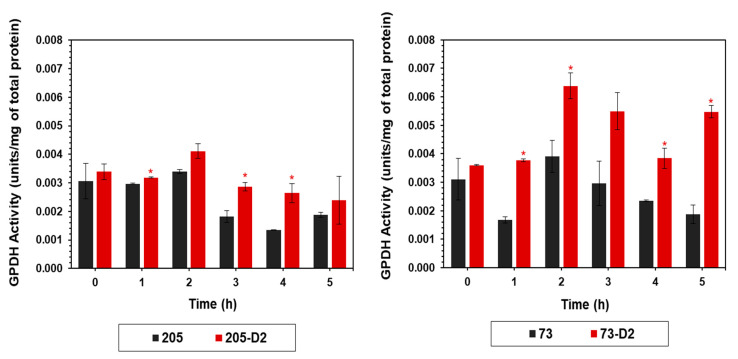
Temporal quantitation of GPDH activity of *S. oralis* strains 73-D2 and 205-D2 vs. their respective *S. oralis* parental strains. GPDH activity was assayed from cell-free lysates from cultures cultivated in BHI and harvested at multiple time points (0–5 h). The data represent the mean (±SD) of three independent experiments. Statistical significance (*) was assessed by using Student’s *t*-test (*p* ≤ 0.05); * *p* < 0.05, D2 strains vs. their respective HLDR and non-HLDR parental strains.

GPDH serves as a major link between glucose and lipid metabolic pathways (Figure 3). The GPDH activity was substantially increased in DAP-R strain 73-D2 vs its respective parental strain over the same early growth period as noted above (2–5 h) to levels exceeding those seen in DAP-R strain 205-D2 (Figure 5).

#### 4.1.4. Measurement of Acetate and Ammonium in Culture

**Figure 6:**
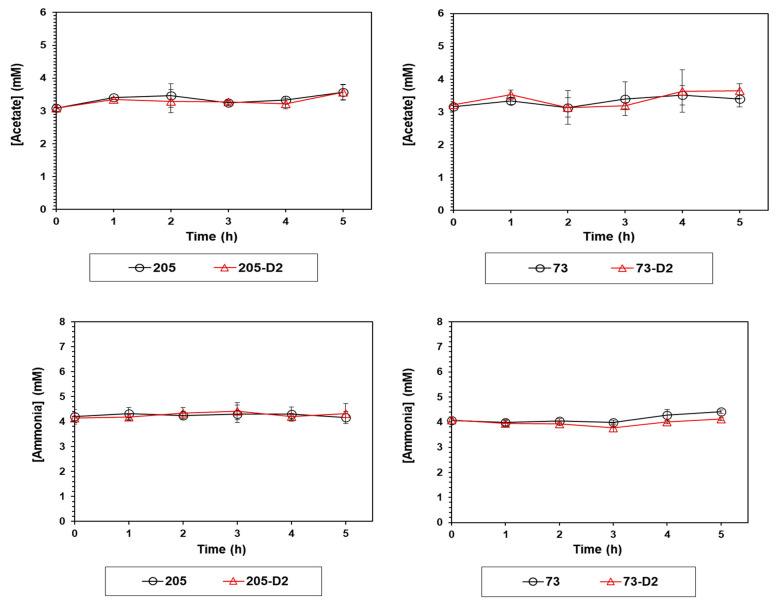
Measurements of acetate and ammonium concentrations in culture media of 73-D2 and 205-D2 vs. their respective *S. oralis* parental strains. Acetate and ammonium concentrations in the culture medium of D2 strains vs. their respective HLDR and non-HLDR parental strains were analyzed hourly. Data represent the mean (±SD) of three independent experiments. Statistical differences for D2 strains vs. their respective HLDR and non-HLDR parental strains were determined by Student’s *t*-test.

We recently showed that selected DAP-R *S. mitis/oralis* strains exhibited significantly altered patterns of acetate and amino acid metabolism [11]. The secretion of acetate and deamination of amino acids can cause the respective accumulation of acetate and ammonium in culture medium during bacterial growth [11,26,28]; moreover, acid accumulation in the medium lowers pH, and can itself inhibit growth [26,28]. Thus, growth media concentrations of acetate and ammonium were measured for both strain-pairs; the accumulation profiles of both acetate and ammonia were unaltered in either strain-pair (Figure 6).

## 5. Genotypic Profiling by WGS

We have previously shown that loss-of-function mutations in key PL biosynthetic genes (such as *cdsA* and *pgsA)* mediate the HLDR phenotype in selected *S. mitis/oralis* strains [8,9,10]. In this investigation, to better understand the genetic correlates of HLDR vs. non-HLDR phenotypes, we performed WGS, comparing D2 passage isolates vs. their respective parental strains (Table 4 and Table 5; Supplementary Table S1). WGS analyses identified mutations in 51 and 57 gene products in the HLDR 73-D2 strain and the non-HLDR 205-D2 strain, respectively, as compared to each parental strain. For example, and relevant to our phenotypic and metabolic metrics, non-synonymous mutations were found in several genes involved in both glucose and PL metabolism in the 73-D2 strain vs. its parental 73 strain (e.g., *ldh* and *pap2* = Thr28Asn, Ile194Val and Ser90Cys, respectively; Table 4). Further, non-HLDR 205-D2 had mutations in genes also involved in PL metabolism (i.e., *gpdh* and *pgsA*), resulting in amino acid changes (i.e., Val137Leu and Ala123del, respectively).

Lastly, genetic mutations were shared in 11 gene products in comparing HLDR 73-D2 and non-HLDR 205-D2 (Supplementary Table S1). Both DAP-R variants shared the same Lys3Glu mutation in a PspC domain-containing protein and a Cys610Gly mutation in a magnesium-translocating P-type ATPase. In two instances, one variant was noted in VGS73-D2, while VGS205-D2 harbors the reversion of that variant. The two proteins predicted as RNA polymerase were β’ subunit (RpoC) and phosphoribulokinase. Their role in DAP-R remains unknown. RpoC mutation was previously described in DAP resistance in a clinical-strain pair of *S. mitis/oralis* and in other Gram-positive pathogens, including *Staphylococcus* spp. [29,30,31] and *Enterococcus faecium* [32,33]. Of note, in the HLDR 73 strain-set, we also observed changes in proteins associated with transcriptional response systems and cell wall biosynthesis (including penicillin-binding proteins) (Table 4). Similarly, many proteins involved in cell wall biosynthesis were also modified in the non-HLDR 205 strain-set. The potential impacts of these latter mutations on any of the phenotypic and/or metabolic metrics observed in this study are unknown at this time.

## 6. Discussion

We and others have described several distinct mechanisms that contribute to the DAP-R phenotype in *S. mitis/oralis* strains [8,9,10,11,34,35]. For example, we previously demonstrated that *S. mitis/oralis* isolates which undergo serial, long-term (6–10 d) in vitro or in vivo passage in DAP exhibit several distinct phenotypic and metabolic signatures, including: (i) disappearance of two key membrane PLs, i.e., PG and CL, with a buildup of the precursor molecule (PA) of the PG-CL biosynthetic pathway (8-10); (ii) increased membrane fluidity; (iii) increased positive surface charge; (iv) selective DAP hyper-accumulation in a minority of streptococcal chain cells; and (v) metabolic perturbations in glucose catabolism [11]. The current study was particularly designed to focus on the early (2 days) impacts and mechanisms of in vitro DAP-passage on two *S. oralis* strains that appear “destined” or not to rapidly evolve either an HLDR or non-HLDR phenotype.

Our current investigation yielded several noteworthy themes. First, similar to other prior HLDR *S. mitis/oralis* strains that we have studied [8,9,10], DAP-R strain 73-D2 exhibited an apparent near-disappearance of PG and CL as compared to its 73 parental strain on both 2D-TLC and lipidomics assays; in contrast, the non-HLDR 205-D2 demonstrated similar PG and CL profiles vs its parental strain. Since negatively charged PG is important for DAP’s “docking site” interactions, and ultimate oligomerization within target CMs [36,37], this PG-CL “disappearance phenotype” appears to be one plausible contributory mechanism for HLDR [8,9,10]. Additionally, negatively charged PLs (such as PG and especially CL) are critical in ‘directing’ DAP’s localization within the cell envelope to its major site of activity; i.e., the septal division plane [8].

Second, apart from the above PL alterations, our lipidomic analyses revealed modifications in several other lipid species, such as DAG, which was increased in the HLDR 73-D2 strain (vs. its parental progenitor). In contrast, the non-HLDR 205 strain-set had similar DAG profiles. These latter observations raise the possibility that the HLDR 73-D2 strain, in order to maintain its overall membrane structure, can efficiently recycle DAG; another possible pathway involved here could involve the PA phosphatase which catalyzes the dephosphorylation of PA to plausibly yield increased DAG in this HLDR strain [38]. It should be also emphasized that there are two key interrelated metabolic pathways which begin from the central metabolite, CDP-DAG: (i) PG phosphate synthase (PgsA), which catalyzes the condensation of glycerol-3-phosphate (G3P) with CDP-DAG, leading to the synthesis of PG phosphate (PGP), the first step in PG biosynthesis; and (ii) then CL synthase (ClsA) catalyzes the synthesis of CL from two molecules of PG (Supplementary Figure S3). PE, PG and CL can, thus, be the ‘building blocks’ for the synthesis of many other lipids [39]. Joyce et al. have recently shown that *S. mitis/oralis* strains can scavenge human metabolites, and use them to synthesize membrane PC, a very rare bacterial PL [40]. Further, our lipidomics analysis suggested a role for other significantly altered lipid species, e.g., Cer, SM, and FFA in 73-D2 and 205-D2 strains as compared to their respective parental strains. Cer, together with SM, can form sphingolipids; even though most pathogenic bacteria are not known to produce sphingolipids, they appear to be capable of using or degrading host sphingolipids to promote their virulence [41]. Unfortunately, the specific virulence roles of PI, PE, PS, PC, Cer, SM, and CE, which were observed in our lipidomics analyses, are poorly understood in bacterial pathogens, including *S. mitis/oralis* strains.

Third, another important feature seen in prior DAP-R *S. mitis/oralis* strains and other DAP-R bacteria (e.g., *S. aureus* and enterococci) is the evolution of substantially increased positive surface charge [8,9,16,24,25]. This adaptation in some DAP-R strains has been postulated to create a more “charge-repulsive milieu” that could contribute to a reduced ability of calcium-DAP to bind to target CMs to initiate its bactericidal effects [8,9,16,24,25]. However, this phenotype was not observed in our HLDR 73 D-2 strain. Thus, the emergence of a “cationic charge-repulsive cell surface” does not appear to be involved in the HLDR phenotype in our HLDR *S. oralis* strain-set.

Fourth, similar to previous observation [8,9,10], the HLDR 73-D2 strain exhibited an early adaptive increase in CM fluidity vs. its parental strain, a phenotype absent in the non-HLDR 205 strain-pair. Fluidity is a key determinant that influences the interactions of cationic antimicrobial peptides (e.g., host defense peptides and calcium-DAP) with the CM, as well as a variety of cellular processes, including the activity of CM-associated enzymes [8,9,16,24,25,42]. Most prior studies point to an ‘optimum’ level of CM fluidity for the interaction of specific cationic antimicrobial peptides (including calcium-DAP) with target bacterial CMs [8,9,10]. These latter data, as well as our current findings, suggest that altered membrane biophysical properties likely contribute to the early evolution of the HLDR phenotype in certain *S. mitis/oralis* strains. Since perturbations in the lipid and PL content can impact CM fluidity, the numerous modifications of these molecules seen above that distinguish the HLDR passage variant from its parental strain are likely in play.

Fifth, to understand the early adaptations in metabolic pathways that underlie the HLDR phenotype, we carried out several targeted metabolic assays to compare the evolution of the HLDR vs. non-HLDR phenotypes in our *S. oralis* strain-sets. Similar to previous observations in other *S. mitis/oralis* strains, the DAP-R 73-D2 strain demonstrated a reduced growth rate profile, especially during early growth phases, as compared to its parental strain; this growth defect was not seen in the 205 strain-pair. This change in the early growth of strain 73-D2 (but not in 205-D2) was associated with decreased glucose utilization as compared to its parental 73 strain. As expected, the accumulation of lactate in the growth media (catalyzed by membrane associated LDH) was largely dependent on the bacterial growth rate and glucose utilization noted above.

*S. mitis/oralis* strains may not completely oxidize glucose to acetyl coenzyme (acetyl-coA) because of their lack of an intact TCA cycle [11]. This can cause acetate to accumulate in the culture medium, associated with decreased media pH [26,28]. Thus, maintenance of the redox status in *S. mitis/oralis* strains must be alternatively performed by other means, e.g., ‘siphoning’ carbon through amino acid biosynthetic pathways [11]. However, in the current study, neither accumulation of acetate nor ammonia were detected early in either DAP-R strain (73-D2 or 205-D2) vs. each’s respective parental strain. These latter data suggested further that glucose metabolism plays the pivotal role in the early transitioning of selected DAP-S *S. mitis/oralis* strains to a stable HLDR phenotype during in vitro DAP exposures.

To further understand the linkage between glucose metabolism and alterations of PL content, we quantified GPDH, since this key enzyme serves as a major link between glucose and lipid metabolism (Supplementary Figure S3). Of note, this enzyme was significantly upregulated early in growth, especially in the HLDR 73-D2 vs its parental 73 strain. The central role of glycerol in intermediary metabolism can have significant pleiotropic effects either in the disappearance of PG and CL or in the accumulation of PA and other lipid species. Taken together, the altered patterns of both glycolytic and lipid metabolites, mediated by membrane-dependent enzymes (such as LDH and GPDH) strongly underscore the pivotal role of these essential cellular pathways in the early evolution of the HLDR phenotype.

Lastly, to elucidate the genetic correlates of glucose and lipid metabolism perturbations identified herein, we performed WGS, which highlighted several mutations in genes involved in lipid and glucose metabolism as correlating with the HLDR phenotype. For example, mutations associated with *pap2* gene were noted in the HLDR 73-D2 strain; this gene encodes a type 2 PA phosphatase (PAP2) enzyme, similar to the phosphatidylglycerophosphatase (PGP) from *E. coli* [43,44]. This phosphatase dephosphorylates PGP to yield PG [43,44]. Another important mutation was found in the *ldh* gene; this might potentially encode for the increased enzymatic activity of LDH we observed in the HLDR 73-D2 strain. Further, a SNP was observed in the *gpdh* gene in the non-HLDR 205-D2 strain that might well connect glucose and lipid metabolism in this strain and also play an important role in the processing of glycerol in lipid metabolism [45]. Moreover, the non-synonymous SNP occurring in *pgsA* in the non-HLDR 205-D2 strain (vs. its parental 205 strain) that mediates the synthesis of PG (and subsequently CL) may be important in maintaining the levels of these key PLs. Collectively, these WGS data suggest that both HLDR and non-HLDR *S. oralis* strains adapt early to DAP exposures by unique genotypic modifications, potentially by both distinct PL biosynthetic modifications, as well as by glycolytic pathway alterations. Of course, it will be important to adjudicate whether these mutations in the above cadre of candidate genes are gain-in-function or loss-in-function perturbations, since both have been described related to DAP-R phenotypes [8,9,10]. The most famous example of this are the gain-in-function mutations in the *mprF* gene of *S. aureus* in DAP-R strains [16,31]. In enterococci, DAP-R has been associated with mutations in two- or three-component regulatory systems (*yycFG*; *liaFSR*), as well as by mutations in *cls* [46,47]. An overall reduction in PLs, including PG, lysyl-PG, and CL, were observed in such DAP-R enterococcal strains [24,48]. However, the most striking feature associated with DAP-R in enterococci is the diversion of anionic PLs (e.g., CL) away from the divisome [49].

As noted above, no distinctive genomic ‘signatures’ were discernable in the WGS analyses above, comparing either the two parental strains or by querying the early (day 2) DAP-passage isolates. Identifying a predictive genomic biomarker(s), exclusively seen in *S. mitis/oralis* parental strains that are ‘destined’ to rapidly evolve an HLDR phenotype when exposed to DAP, has obvious clinical therapeutic implications. However, this will require WGS profiling of a large *S. mitis/oralis* strain cohort, including both HLDR and non-HLDR isolates and ultimately featuring GWAS analytics. Of interest, we have recently reported an experimental IE study in which two *S. mitis/oralis* strains (both possessing the HLDR phenotype in vitro) were treated in vivo with DAP alone or DAP combined with ceftriaxone. As predicted, DAP treatment regimens encompassing human-equivalent standard-to-high dose regimens of 6-12 mg/kg/d, respectively, were ineffective at significantly reducing *S. mitis/oralis* bioburdens in any target tissues (i.e., cardiac vegetations, kidneys, and spleen) [50]. In contrast, combining DAP with ceftriaxone significantly reduced such bioburdens in all target tissues. Additional in vivo investigations of *S. mitis/oralis* strains which are either destined or not to develop the HLDR phenotype in vitro will be required to validate the translational importance of this phenotype.

In conclusion, it appears likely that the early evolution of HLDR in *S. mitis/oralis* strains may occur via a variety of phenotypic, metabolic, and genotypic mechanisms [8,9,10,11]. However, the current study had some important limitations: (i) we only studied two *S. mitis/oralis* strains, one each demonstrating either an early-onset of the “HLDR’’ phenotype or not; these same analytics need to be confirmed in a larger *S. mitis/oralis* strain cohort; (ii) we only examined phenotypic, metabolic, and genotypic parameters which occurred early post-DAP exposures; these parameters need to be further compared during later DAP exposure times; (iii) our comparative metrics compared outcomes after in vitro DAP exposures; we plan on carrying out additional DAP exposure profiling in in vivo DAP-exposed *S. mitis/oralis* strains, such as from the experimental IE model [50] (iv) other parameters of DAP:CM interactions associated with DAP-R need to be interrogated (e.g., localization of CL at the septal divisome [8,9] or hyper-accumulation of DAP within DAP-R cell subpopulations [8]); and (v) although WGS was performed, follow-up investigations of gene expression differences (e.g., RNA sequencing) need to be carried out.

## Figures and Tables

**Table 1:** DAP MICs of the study strains.

	*S. oralis* Strains	DAP MIC (µg/mL)	DAP MIC (Passaged in DAP Free Media for 5 Days)
	73	0.5	0.5
**HLDR**	73-D2	>256 *	>256
	205	0.5	0.5
**Non-HLDR**	205-D2	8 **	2

* Stable DAP MIC in passage in DAP-free media; ** Unstable DAP MIC in passage in DAP-free media.

**Table 2:** Cell membrane fluidity and surface charge of study strains.

*S. oralis* Strains	CM Fluidity: PI Value (Mean ± SD)	Surface Charge: % of Cyt C in Supernatant (Mean ± SD)	BODIPY-DAP Binding: Fluorescence Intensity (Mean ± SD)
**73**	0.298 ± 0.01	76 ± 3	553 ± 28
**73-D2**	0.271 ± 0.01 *	61 ± 1 *	592 ± 17 *
**205**	0.268 ± 0.01	62 ± 8	405 ± 3
**205-D2**	0.269 ± 0.01	58 ± 7	430 ± 24

Data represent the mean (±SD) of three independent experiments on different days. Statistical significance (*) for D2 strains relative to their HLDR and non-HLDR parental strains were assessed using Student’s *t*-test (*p* ≤ 0.05). * *p* < 0.05 D2 vs. respective parental strains. An increased amount of cytC in the supernatant correlates to a greater positively charged bacterial surface; a lower PI value equates to a higher degree of CM fluidity.

**Table 3:** Quantification of phospholipid (PL) composition of study strains.

	% of PL Species (Mean ± SD)		
**Strains**	**PG**	**CL**	**PA**
**73**	18 ± 8	52 ± 4	31 ± 10
**73-D2**	0 ± 0	0 ± 0	100 ± 0 *
**205**	29 ± 22	67 ± 19	4 ± 4
**205-D2**	18 ± 7	68 ± 6	15 ± 5 *

Data represent the mean (±SD) of three independent experiments. Statistical differences for D2 strains relative to their HLDR and non-HLDR parental strains were determined by Student’s *t*-test; * *p* < 0.05 D2 vs. respective parental strains. PG= phosphatidylglycerol; CL = cardiolipin; PA = phosphatidic acid.

**Table 4:** Summary of amino acid changes (*n* = 53) in 73-D2 versus parental 73 *S. oralis*.

Predicted Amino Acid Change ^a^	Predicted Protein Function	Gene ID
Val876Ile	LPXTG-anchored beta-N-acetylhexosaminidase StrH	gene-EL140_RS00270
Lys3Glu	PspC domain-containing protein	gene-EL140_RS00510
Lys175Gly	DUF1307 domain-containing protein	gene-EL140_RS02430
Lys144Gln	response regulator transcription factor VncR	gene-EL140_RS02580
Thr131Ser, Lys135Val	rRNA pseudouridine synthase	gene-EL140_RS01050
Lys320Asn	glucosaminidase domain-containing protein	gene-EL140_RS03645
2_5delATGGTinsTGGCa, Lys6Gln	tagatose-bisphosphate aldolase	gene-EL140_RS04205
Arg518Ser, Val527Leu	glycoside hydrolase family 13 protein	gene-EL140_RS04725
Ile131Val	ABC transporter permease	gene-EL140_RS00390
Ile27Met	hypothetical protein	gene-EL140_RS00190
Thr28Asn, Ile194Val	L-lactate dehydrogenase	gene-EL140_RS05540
Ser134Gly	hypothetical protein	gene-EL140_RS00190
66delAa	Fe-S cluster assembly protein SufB	gene-EL140_RS05570
Lys1240Asp	Cna B-type domain-containing protein	gene-EL140_RS05785
Gly71fs	YdbC family protein	gene-EL140_RS05795
Ser522Leu	ABC transporter ATP-binding protein/permease	gene-EL140_RS00650
Gln569Glu	aminodeoxychorismate synthase component I	gene-EL140_RS06465
Leu60Pro	response regulator	gene-EL140_RS06480
Ser242Thr	ammonia-dependent NAD(+) synthetase	gene-EL140_RS06505
Leu417Gln	YSIRK-type signal peptide-containing protein	gene-EL140_RS06920
Val144Ala	MarR family transcriptional regulator	gene-EL140_RS01740
2652delGACATinsAACACa	valine—tRNA ligase	gene-EL140_RS07795
Ile1380Thr	YSIRK-type signal peptide-containing protein	gene-EL140_RS06920
GlnAla68GluLeu	phosphoribulokinase	gene-EL140_RS08745
Cys610Gly	magnesium-translocating P-type ATPase	gene-EL140_RS08825
Cys104Ser	GH92 family glycosyl hydrolase	gene-EL140_RS09195
TyrLys427PheGlu	cell surface protein	gene-EL140_RS09325
Ala900Gly, Ala918Val, Ala912Val, Phe461Leu, Asp456Asn	LPXTG cell wall anchor domain-containing protein	gene-EL140_RS06375
Asn82Ser	DUF6261 family protein	gene-EL140_RS09590
Lys292Asn, Ala298Gln	LPXTG cell wall anchor domain-containing protein	gene-EL140_RS06375
Ile99Val, Ala249Ser	methyltransferase domain-containing protein	gene-EL140_RS00590
Lys192Asn	hemolysin family protein	gene-EL140_RS01300
Thr68Met	endonuclease MutS2	gene-EL140_RS01700
Met44Val	hypothetical protein	gene-EL140_RS00190
Ile166Ser	N-acetylmuramoyl-L-alanine amidase	gene-EL140_RS02630
Arg68Gly, Glu72Asp	GDSL-type esterase/lipase family protein	gene-EL140_RS03180
His4Tyr	Nramp family divalent metal transporter	gene-EL140_RS03335
ArgLysGly154LysAlaGlu, Glu308fs, Ala2Val, Gln6Asn	tagatose-6-phosphate kinase	gene-EL140_RS04200
Asn240Asp	pneumococcal-type histidine triad protein	gene-EL140_RS04260
Asp61Tyr, Val70Ala	HNH endonuclease	gene-EL140_RS04615
Leu229Ile	hypothetical protein	gene-EL140_RS00190
Phe125Leu, Thr105Pro	hypothetical protein	gene-EL140_RS00190
Ile110Met	nicotinate phosphoribosyltransferase	gene-EL140_RS06510
Ser755Thr, Gln681Glu	G5 domain-containing protein	gene-EL140_RS06930
Asn624Glu	penicillin-binding protein PBP2B	gene-EL140_RS07010
Asn861Ser	antigen I/II family LPXTG-anchored adhesin	gene-EL140_RS07990
Thr33Ala, Asp35Gly	ABC transporter permease	gene-EL140_RS00390
Pro1264Leu	glycoside hydrolase N-terminal domain-containing protein	gene-EL140_RS08115
Glu379Asp	penicillin-binding protein PBP2X	gene-EL140_RS08125
Ser90Cys	phosphatase PAP2 family protein	gene-EL140_RS01360
Glu871Asp	DNA-directed RNA polymerase subunit beta’	gene-EL140_RS08440

^a^: Number refers to nucleotide position; fs—frameshift.

**Table 5:** Summary of amino acid changes (*n* = 64) in 205-D2 vs. parental 205 *S. oralis*.

Predicted Amino Acid Change ^a^	Predicted Protein Function	Gene ID
Glu144Gln	tagatose-bisphosphate aldolase	gene-EL140_RS04205
Glu458Asp	fibronectin-binding SSURE repeat-containing protein	gene-EL140_RS00420
Lys3Glu	PspC domain-containing protein	gene-EL140_RS00510
Met6Leu	ribonuclease M5	gene-EL140_RS01180
Asn331Gln, Val2276Leu, His/Lys331Gln	accessory Sec-dependent serine-rich glycoprotein adhesin	gene-EL140_RS01855
leu51Met, Ser151Ala, Arg165Lys	serine O-acetyltransferase	gene-EL140_RS02135
Asn8Asp	nucleoside phosphorylase	gene-EL140_RS00405
Asp43Glu	DUF1307 domain-containing protein	gene-EL140_RS02430
Arg6Trp	DUF6287 domain-containing protein	gene-EL140_RS02620
599insAT	N-acetylmuramoyl-L-alanine amidase	gene-EL140_RS02630
Ile53Val, His139Arg, Ser690Phe, Leu694Val	heavy metal translocating P-type ATPase	gene-EL140_RS00600
Asn295Ser	hypothetical protein	gene-EL140_RS00190
Gln992His	carbamoyl-phosphate synthase large subunit	gene-EL140_RS03330
Glu438ins, GluGlnPro439HisProThr	DNA primase	gene-EL140_RS03860
Arg51His, AspGln566GlyLeu, Val570Ala	glycoside hydrolase family 13 protein	gene-EL140_RS04725
Glu229Lys	YjjG family noncanonical pyrimidine nucleotidase	gene-EL140_RS04975
Asn142Asp	SUF system NifU family Fe-S cluster assembly protein	gene-EL140_RS05575
Ala17Val	aminoglycoside 6-adenylyltransferase	gene-EL140_RS05640
Phe93Cys	VanZ family protein	gene-EL140_RS05855
Glu47Lys	UPF0223 family protein	gene-EL140_RS06150
Asp65Glu	SDR family oxidoreductase	gene-EL140_RS06415
Val294Ile	ribonuclease Z	gene-EL140_RS06420
LeuAla83LeuThr, GlyLeu86AlaIle	hypothetical protein	gene-EL140_RS00190
Asn350Lys	G5 domain-containing protein	gene-EL140_RS06930
Ser824Asn, Thr1012Asn, Thr843Ile, Thr820Asn	G5 domain-containing protein	gene-EL140_RS06930
Val1055Ala	exo-alpha-sialidase	gene-EL140_RS07065
Val138Met	DUF421 domain-containing protein	gene-EL140_RS07435
Asp1472Glu	glycoside hydrolase N-terminal domain-containing protein	gene-EL140_RS08115
Asp871Glu	DNA-directed RNA polymerase subunit beta’	gene-EL140_RS08440
Cys610Gly	magnesium-translocating P-type ATPase	gene-EL140_RS08825
Met267Ile	D-alanyl-lipoteichoic acid biosynthesis protein DltD	gene-EL140_RS09230
Arg74Ser	hypothetical protein	gene-EL140_RS00190
Ala862Thr	LPXTG cell wall anchor domain-containing protein	gene-EL140_RS06375
Val137Leu	NAD(P)H-dependent glycerol-3-phosphate dehydrogenase	gene-EL140_RS00645
Thr718Ala	HAD-IC family P-type ATPase	gene-EL140_RS01060
LysValLeuValGly150AsnAlaLeuThrIle, Ala159Ser	sugar transferase	gene-EL140_RS01435
IleAspLeu211ValAlaIle	metal ABC transporter ATP-binding protein	gene-EL140_RS02210
Asn117Ser	DNA/RNA non-specific endonuclease	gene-EL140_RS01295
Leu6Gln	NAD-dependent protein deacylase	gene-EL140_RS03745
Asp74His	nucleotidyltransferase	gene-EL140_RS01975
Gly14Ala	MBL fold metallo-hydrolase	gene-EL140_RS02220
Lys12Gln	MmcQ/YjbR family DNA-binding protein	gene-EL140_RS05435
Ala177Val	DNA topology modulation protein	gene-EL140_RS05725
AsnLeu84ThrIle	prephenate dehydrogenase	gene-EL140_RS06020
Asn78Ser, Thr72Ala	inositol monophosphatase family protein	gene-EL140_RS06145
His772Asn, Asn770Tyr, Arg768Gln	Ltp family lipoprotein	gene-EL140_RS06460
AlaAspVal352ValAlaIle	sensor histidine kinase	gene-EL140_RS01655
Val24Glu	response regulator	gene-EL140_RS06480
Phe200Tyr	Nif3-like dinuclear metal center hexameric protein	gene-EL140_RS06860
Ter295Lysext ^a,^*	ROK family protein	gene-EL140_RS02070
Ala120Val, ThrIle109LeuLeu	ABC transporter permease	gene-EL140_RS00390
Ser1567Thr, GlyThrGlyAla1549SerThrAsnVal	YSIRK-type signal peptide-containing protein	gene-EL140_RS06920
Ala22Thr	50S ribosomal protein L23	gene-EL140_RS08720
GluLeu68GlnAla, Val65Ile, Asn60Glu	phosphoribulokinase	gene-EL140_RS08745
Ala123del	CDP-diacylglycerol—glycerol-3-phosphate 3-phosphatidyltransferase	gene-EL140_RS09460
Pro15Leu	AraC family transcriptional regulator	gene-EL140_RS05010
Met1fs	hypothetical protein	gene-EL140_RS00190

^a^: Numbers refer to amino acid position; * protein sequence extension; fs—frameshift.

## Data Availability

Data is contained within the article and Supplementary Material.

## Supplementary Materials

The following supporting information can be downloaded at: https://www.mdpi.com/article/10.3390/antibiotics12071083/s1, Table S1. Summary of gene product changes seen in both *S. oralis* D2 derivatives compared to its D0 parental strains. Figure S1. Phospholipid (PL) patterns of HLDR and non-HLDR strains vs. respective parental strains. Figure S2 (A). Lipidomic data of 73-D2 and 205-D2 *S. oralis* strains vs. their respective parental strains; (B) Lipidomic data of 73 -D2 and 205-D2 vs. their respective parental strains; (C) Lipidomic data of 73-D2 and 205-D2 vs. their respective parental strains. Figure S3. Summary of linkage of glycolysis and phospholipid pathways.
