# Supplementary material for: Membrane Phenotypic, Metabolic and Genotypic Adaptations of Streptococcus oralis Strains Destined to Rapidly Develop Stable, High-Level Daptomycin Resistance during Daptomycin Exposures
Source: Antibiotics (Basel). 2023 Jun 21;12(7):1083. doi: 10.3390/antibiotics12071083 (PMC10376253; doi:10.3390/antibiotics12071083)
Supplement: Supplementary file 1 [file antibiotics-12-01083-s001.zip › antibiotics-2440383-supplementary.pdf]

Supplementary Materials

Membrane Phenotypic, Metabolic and Genotypic Adaptations of *Streptococcus oralis* Strains Destined to Rapidly Develop Stable, High-Level Daptomycin Resistance during Daptomycin Exposures

Nagendra N. Mishra [1,2,*], Rodrigo de Paula Baptista [3,4,5], Truc T. Tran [3,4,5], Christian K. Lapitan [1], Cristina Garcia-de-la-Maria [6,7], Jose M. Miró [7], Richard A. Proctor [8] and Arnold S. Bayer [1,2]

[1] Division of Infectious Diseases, The Lundquist Institute at Harbor-UCLA Medical Center, 1124 West Carson St. MRL Bldg. Room 224, Torrance, CA 90502, USA
[2] The David Geffen School of Medicine, University of California, Los Angeles (UCLA), Los Angeles, CA 90095, USA
[3] Center for Infectious Diseases, Houston Methodist Research Institute, Houston, TX 77030, USA
[4] Division of Infectious Diseases, Department of Medicine, Houston Methodist Hospital, Houston, TX 77030, USA
[5] Department of Medicine, Weill-Cornell Medical College, New York, NY 10065, USA
[6] Infectious Diseases Service, Hospital Clinic—IDIBAPS, University of Barcelona, 08036 Barcelona, Spain;
[7] CIBERINFEC, Instituto de Salud Carlos III, 28220 Madrid, Spain
[8] The Department of Medicine, University of Wisconsin School of Medicine, Madison, WI 53705, USA
* Correspondence: nmishra@lundquist.org or nnmishra@ucla.edu; Tel.: +1-310-222-4013; Fax: +1-310-803-5620

Table S1. Summary of gene product changes seen in both *S. oralis* D2 derivatives compared to its D0 parental strains.

Predicted changes in 73-D2	Predicted changes in 205-D2	Predicted protein function
Lys3Glu	Lys3Glu	PspC domain-containing protein
Ile166Ser	599insAT	N-acetylmuramoyl-L-alanine amidase
Arg518Ser, Val527Leu	Arg51His, AspGln566GlyLeu, Val570Ala	glycoside hydrolase family 13 protein
Ser755Thr, Gln681Glu	Ser824Asn, Thr1012Asn, Thr843Ile, Thr820Asn	G5 domain-containing protein
Pro1264Leu	Asp1472Glu	glycoside hydrolase N-terminal domain-containing protein
Glu871Asp	Asp871Glu	DNA-directed RNA polymerase subunit beta'
Cys610Gly	Cys610Gly	magnesium-translocating P-type ATPase
Ala900Gly, Ala918Val, Ala912Val, Phe461Leu, Asp456Asn	Ala862Thr	LPXTG cell wall anchor domain-containing protein
Leu60Pro	Val24Glu	response regulator
Ile1380Thr	Ser1567Thr, GlyThrGlyAla1549SerThrAsnVal	YSIRK-type signal peptide-containing protein
GlnAla68GluLeu	GluLeu68GlnAla, Val65Ile, Asn60Glu	phosphoribulokinase

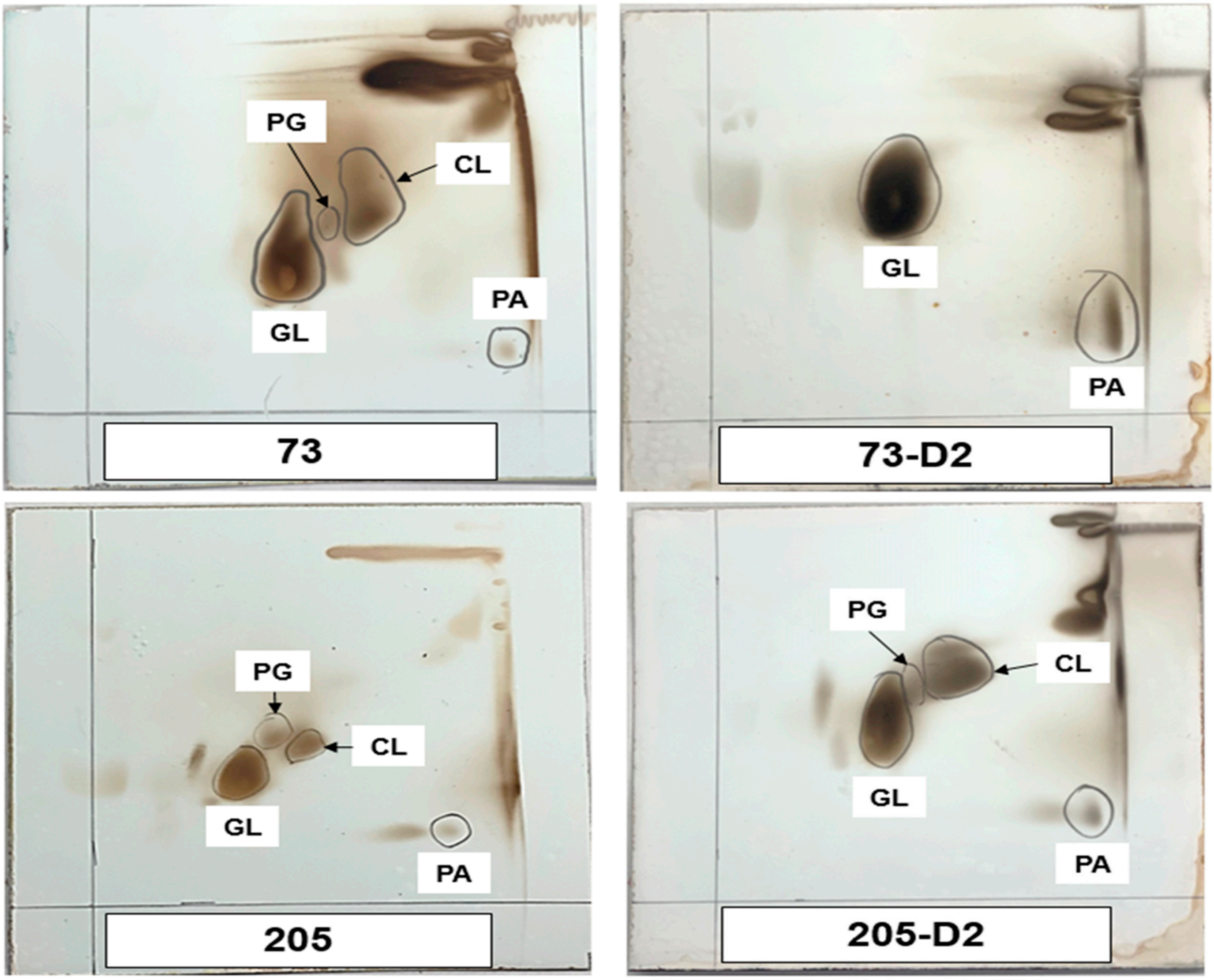

Figure S1. Phospholipid (PL) patterns of HLDR and non HLDR strains vs. respective parental strains. The PL patterns of phosphatidyl glycerol (**PG**), cardiolipin (**CL**), and phosphatidic acid (**PA**) including glycolipid (**GL**) were consistent in each experiments.

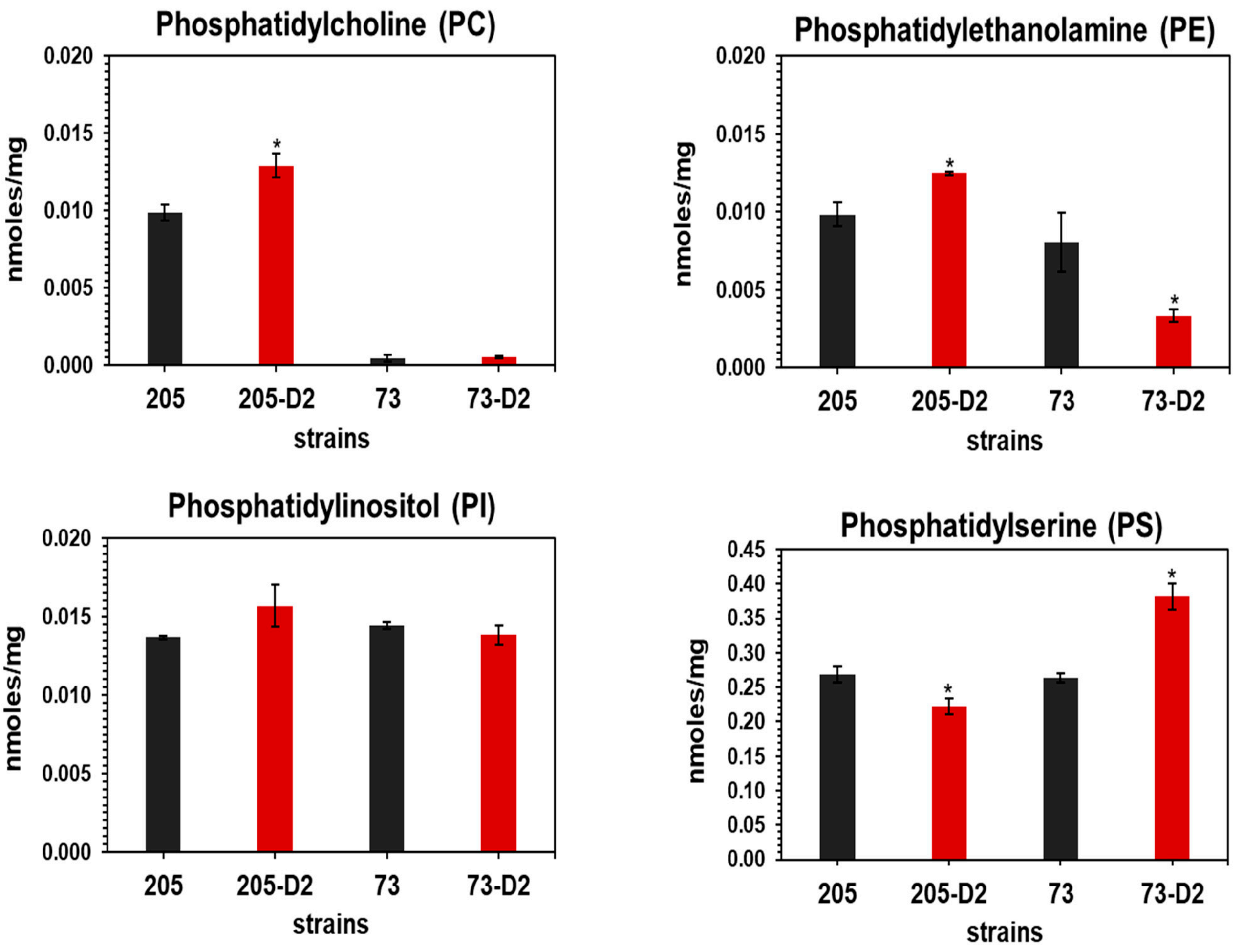

Figure S2A. Lipidomics data of 73-D2 and 205-D2 *S. oralis* strains vs. their respective parental strains. These data represent the mean (+/- SD) of three independent experimental runs from different lipid extracts. Statistical differences for D2 strains relative to their HLDR and non-HLDR parental strains were carried out by Student's t-test; *$p<0.05$ Parental strains vs. D2 strains.

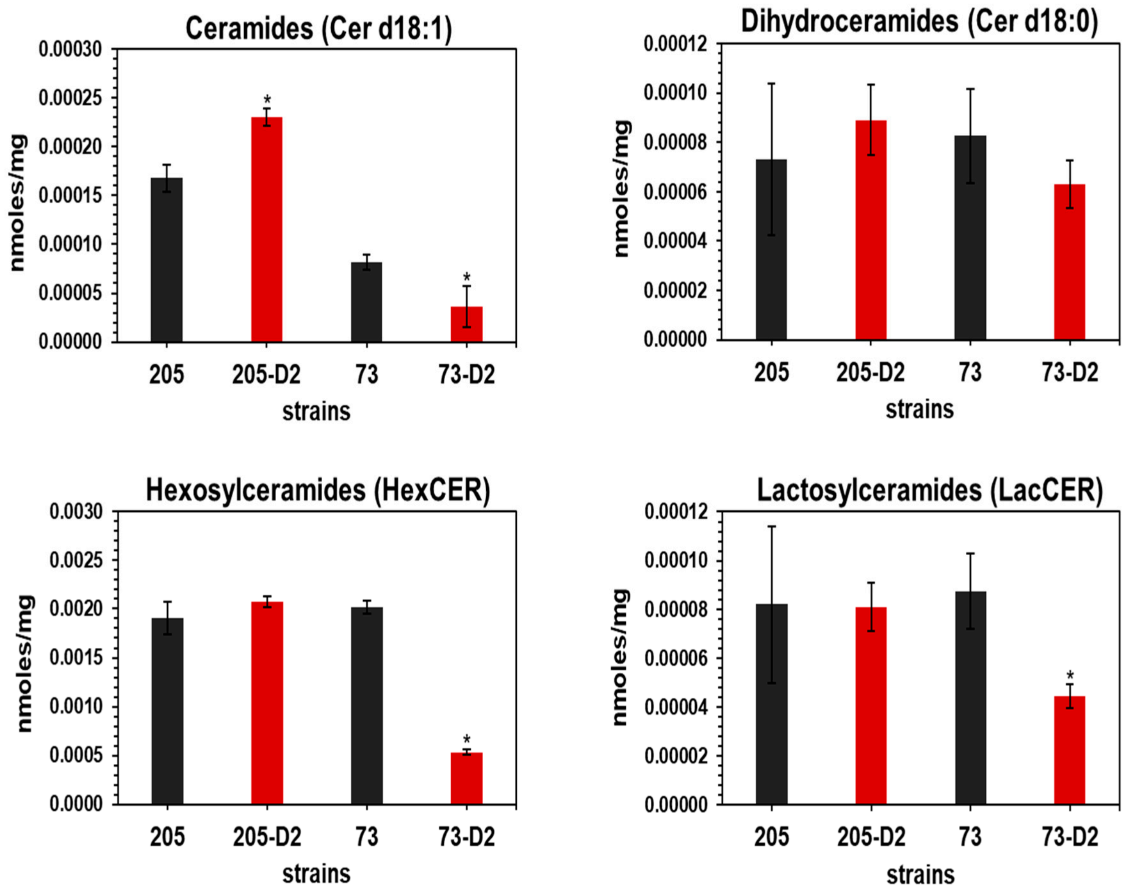

Figure S2B. Lipidomics data of 73 -D2 and 205-D2 vs. their respective parental strains, Data represent the mean (+/- SD) of three different experiments from various lipid extracts. Statistical differences for D2 strains relative to their HLDR and non-HLDR parental strains were analyzed by Student's t-test; *p<0.05 Parental strains vs. D2 strains.

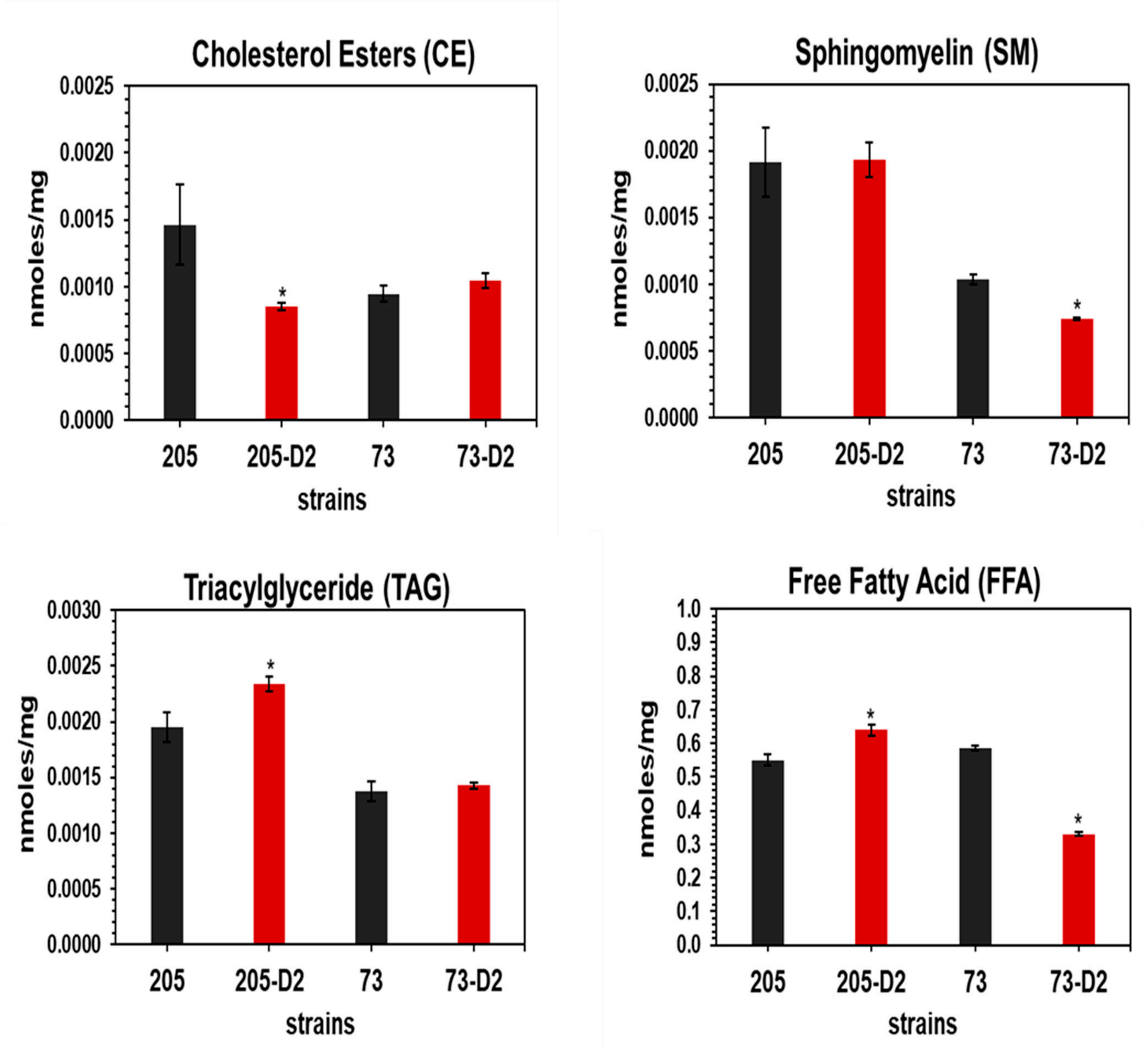

Figure S2C. Lipidomics data of 73-D2 and 205-D2 vs. their respective parental strains. Data represent the mean (+/- SD) of three independent experiment from various lipid extracts. Statistical differences for D2 strains relative to their HLDR and non-HLDR parental strains were demonstrated by Student's t-test; *$p<0.05$ Parental strains vs. D2 strains.

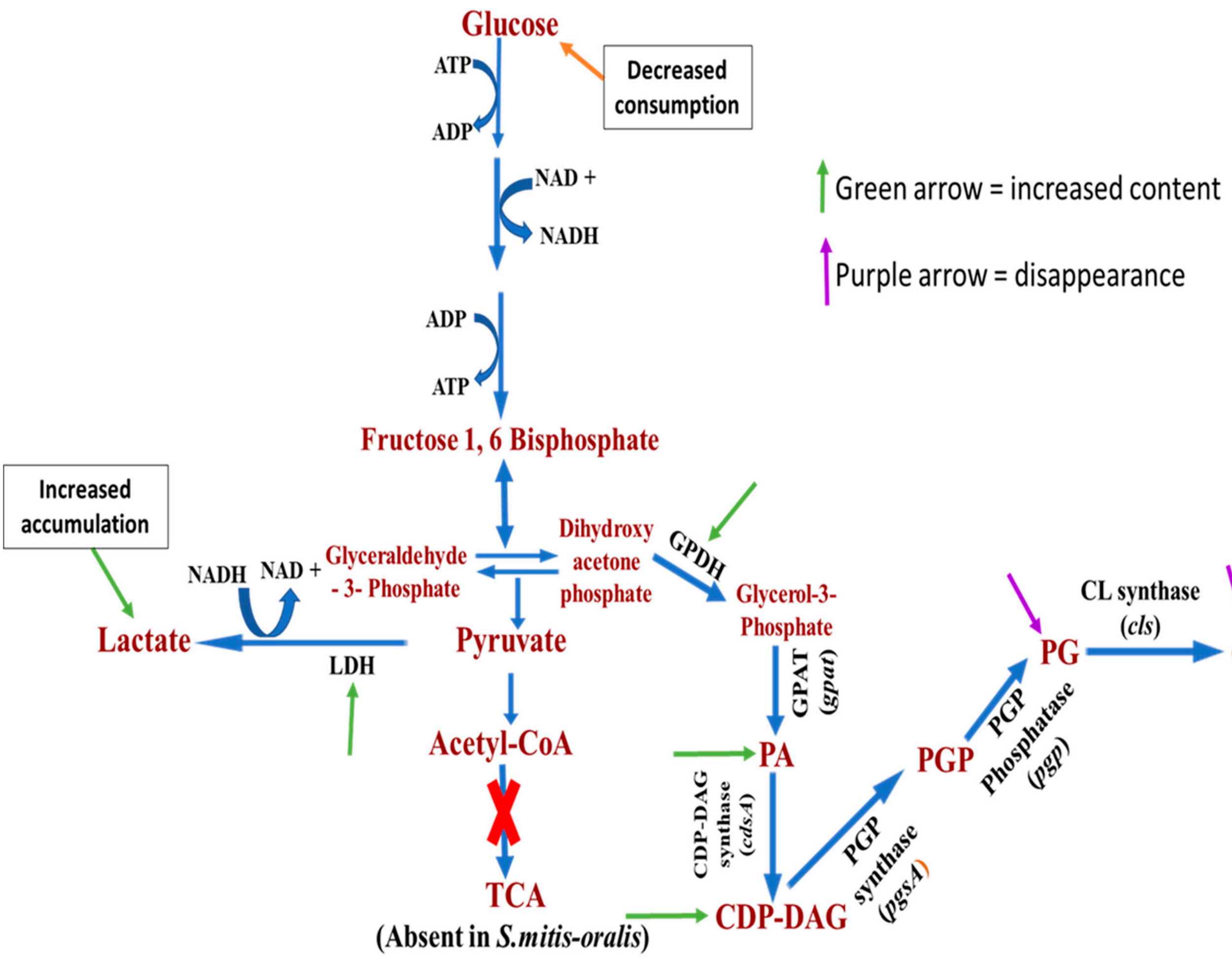

Figure S3. Summary of linkage of Glycolysis and phospholipid pathways. Arrows indicated the concentrations of metabolites or activity of enzymes were decreased or increased in HLDR 73-D2 strain vs. 73 parental strain. PGP (phosphatidylglycerol phosphate); CDP-DAG (Cytidine diphosphate diacylglycerol); GPDH (glycerol-3-phosphate dehydrogenase); GPAT (Glycerol-3-phosphate acyltransferase); LDH (lactate dehydrogenase); TCA (Tricarboxylic acid) cycle.
